# Characterization of Sicilian Honeys Pollen Profiles Using a Commercial E-Tongue and Melissopalynological Analysis for Rapid Screening: A Pilot Study

**DOI:** 10.3390/s18114065

**Published:** 2018-11-21

**Authors:** Ambra R. Di Rosa, Anna M. F. Marino, Francesco Leone, Giuseppe G. Corpina, Renato P. Giunta, Vincenzo Chiofalo

**Affiliations:** 1 Dipartimento di Scienze Veterinarie, Università degli Studi di Messina, 98168 Messina, Italy; francesco.leone190@gmail.com; 2 Istituto Zooprofilattico Sperimentale della Sicilia, 95125 Catania, Italy; annamaria.marino@izssicilia.it (A.M.F.M.); giuseppe.corpina@izssicilia.it (G.G.C.); renato.giunta@izssicilia.it (R.P.G.); 3 Dipartimento di Scienze Chimiche, Biologiche, Farmaceutiche ed Ambientali, Università degli Studi di Messina, 98167 Messina, Italy; vincenzo.chiofalo@unime.it

**Keywords:** honey, artificial senses, E-tongue, melissopalynological analysis, data fusion

## Abstract

Honey is usually classified as “unifloral” or “multifloral”, depending on whether a dominating pollen grain, originating from only one particular plant, or no dominant pollen type in the sample is found. Unifloral honeys are usually more expensive and appreciated than multifloral honeys, which highlights the importance of honey authenticity. Melissopalynological analysis is used to identify the botanical origin of honey, counting down the number of pollens grains of a honey sample, and calculating the respective percentages of the nectariferous pollens. In addition, sensory properties are also very important for honey characterization, and electronic senses emerged as useful tools for honey authentication. In this work, a comparison of the results obtained from melissopalynological analysis with those provided by a potentiometric electronic tongue is given, resulting in a 100% match between the two techniques.

## 1. Introduction

Honey is defined as the natural sweet substance produced by *Apis mellifera* bees from the nectar of plants, from secretions of living parts of plants or from excretions of plant-sucking insects on plants. Bees collect it, transform it by combining it with specific substances of their own, deposit it, dehydrate it, store it and leave it in honeycombs to ripen and mature [1,2,3,4,5]. Honey is a very nutritious food product, being a solution of sugars, mainly fructose and glucose, with a small amount of higher sugars, enzymes, acids, salts and aromatic substances [6,7,8]. Its composition depends on several factors, such as the floral source used to collect the nectar, the environment where the plants grow, and the insect itself [9,10]. Honeys may be classified as “unifloral” or “multifloral”, depending on whether a dominating pollen grain, originated from only one particular plant or no dominant pollen type in the sample is found. Unifloral honeys are usually more expensive and appreciated than multifloral honeys [11,12,13,14,15,16]. In addition, the high variability of this product highlights the importance of honey authenticity, which is a fundamental requirement for all food products that can easily be adulterated [17]. Traditionally, melissopalynological analysis is used to identify the botanical origin of honey. This approach consists of counting down the number of pollens grains in a honey sample, and calculating the respective percentages of the nectariferous pollens [16,18]. Sensory properties are also very important for honey characterization, and sensory analyses represent an important complementary part of the pollen analysis [19,20,21]. However, it always requires a panel of skilled assessors [22]. In addition, analytical and quantitative methods such as high-performance liquid chromatography (HPLC) and high-performance anion-exchange chromatography are also routinely performed, resulting in a discrimination obtained from the general fingerprints or from the different profiles of the components identified (e.g., amino acids, benzene derivatives, terpenes) [23,24,25,26,27,28,29]. However, these methods are laborious and time-consuming, require considerable analytical skills, involve a lot of tedious and complex pre-treatment of samples, and use many hazardous organic reagents that require high costs for storage and disposal [30]. For this reason, the growing interest toward electronic senses [31,32] coupled with chemometrics, lead to successful application in the field of honey authentication [33]. The E-tongue is a device comprising an array of non-specific, poorly selective chemical sensors, with partial specificity, which operates in an aqueous environment, for the recognition of qualitative and quantitative composition of multispecies solutions [34]. In principle, the sensor array of an E-tongue produces a pattern of signals, which is correlated to certain features or qualities of the sample [35,36], and processed with multivariate statistical analysis [37,38]. Regarding the sensor array used in the design of E-tongues, a wide variety of chemical sensors have been employed: electrochemical, optical, mass, and enzymatic [36,39,40,41]. Features of these sensors are different from those of traditional chemical sensors. Instead of a high selectivity in a substance detection, they have an overall selectivity that provides for global information on the solution, which is later applied as a digital fingerprint of the gustatory compounds [36]. Ion-selective electrodes (ISEs) represent the largest group among potentiometric sensors [36,39,42]. The principle of operation is based on the measurement of the potentials of non-polarized electrodes lacking current flow, which is a function of the activity of the ionic species in the sample solution, and it is formed at the ion-sensitive membrane, where the selective complexation and ion recognition of the analyte molecules occurs [36,41]. Voltammetric sensors are also extensively used in E-tongue systems [39,41]. In voltammetric techniques, a potential is applied on the working electrode, followed by the measurement of the resulting current between the working electrode and the reference electrode. As a result, an electrochemical redox reaction occurs at the electrodes’ surface, and gives rise to the measured current [36]. So far, E-tongues have been successfully used for honey botanical origin identification, providing an economic and rapid method of recognition [12,16,25,43,44,45,46,47]. In particular, E-tongues have been used to classify honey according to its botanical [8,43,47,48] or geographical origin [43,49], and to detect adulterations with sugar syrups [8,50,51]. In addition, a potentiometric E-tongue has been used to perform a “human-like” sensory evaluation of honey, based on the correlation of the E-tongue’ sensor scores with sugars content and pH values, as they have the major influence on the sweetness, bitterness and sourness of the honey [47]. Scepankova et. al. [52] studied the effects of high pressure and temperature on heather honey during storage. Italy and its islands, including mainly Sicily, have a strong vocation for honey production. In recent years, however, the climatic trend has severely penalized Sicily and, for example, the production of citrus honey, particularly appreciated by the market, has been scarce and often other mixed nectars have been found (ONM, 2018). For this reason, the beekeepers have strongly urged the choice of a method to be able to make a first screening of the botanical origin of the honey produced, in order to make quick strategic choices during production, without waiting for the results of official analyzes (melissopalynological analysis). So, the aim of the present work was to validate the results obtained by a potentiometric E-tongue with those provided from the pollen analysis, in order to develop a simple approach, involving a single instrument, with no sample preparation and low cost of the analysis, which can be performed by the manufacturers themselves. For this reason, our attention was focused on a commercial E-tongue, and on four honey varieties, being the most produced in Sicily.

## 2. Materials and Methods

### 2.1. Honey Samples

**Figure 1:**
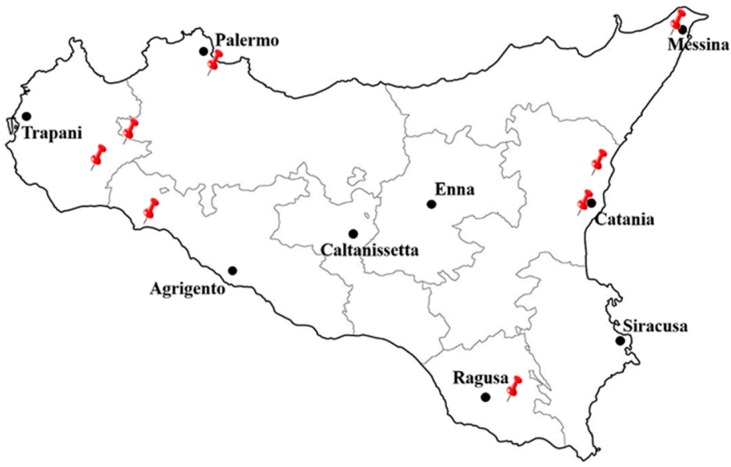
Drawing pins indicates the areas from where the honey samples have been acquired.

In the present study, twenty-three unifloral honey samples, coming from different areas of Sicily (Figure 1), have been used.

Four of them, that is, 2 Chestnut honeys and 2 Citrus honeys, were acquired through participation in the BIPEA (Bureau InterProfessionnel d’Etudes Analytiques) Proficiency Testing, opened to laboratories from 120 countries worldwide. Therefore, the results obtained from these samples are supported by the final reports from the organizer. On the other hand, the remaining 19 samples were kindly supplied from local manufacturers, which provided reliable information on the honeys, including the production source. In particular, 5 were Chestnut honeys (*Castanea sativa*), 6 Eucalyptus honeys (*Eucalyptus camaldulensis* and *Eucalyptus occidentalis*), 6 Sulla honeys (*Hedysarum coronarium*) and 2 Citrus honeys (*Citrus* spp.). These varieties have been chosen due to the presence of normative requirements, establishing the identification parameters and the analytical methods for the definition of “Eucalyptus honeys” (UNI 11383:2010), “Chestnut honeys” (UNI 11376:2010) and “Citrus honeys” (UNI 11384:2010) [53,54,55]. Moreover, for all the varieties used, the identification characteristics are well harmonised in the international literature, the recognition is simple, the pollen grains percentage content is high, and the sensory attributes are well defined. Table 1 resumes the sample set.

### 2.2. E-Tongue

In the present work, a potentiometric E-tongue (αAstree, Alpha M.O.S., Toulouse, France), equipped with seven potentiometric sensors designated as JB, BA, BB, HA, ZZ, CA and GA by the manufacturer (Alpha M.O.S.), an Ag/AgCl reference electrode (Metrohm, Ltd., Herisau, Switzerland), a mechanical stirrer (Metrohm, Ltd.), a 48-position Sample Changer and a 759 Swing Head for sampling (Metrohm, Ltd.), an interface electronic module for signal amplification and analog to digital conversion (Alpha M.O.S.) has been employed. The electronic tongue was connected to a personal computer with the Astree II software (Alpha M.O.S., Version 12.4., 2012) installed. The software automatically gathers and stores the outputs of the sensors. The sensors used in this study are chemically sensitive field-effect transistors (chemFET). The sensors were specially designed by the manufacturer for food and beverage analysis (αAstree User’s Manual Alpha M.O.S., 2012). Before starting samples analysis, conditioning, calibration and diagnostic steps were performed: for conditioning and calibration a solution of hydrochloric acid (0.01 mol/L) was used, while for diagnostic step 3 solutions were used, one of hydrochloric acid (0.01 mol/L), one of sodium glutamate (0.01 mol/L) and one of sodium chloride (0.01 mol/L) (αAstree User’s Manual Alpha M.O.S., Toulouse (2012)). Then the samples array was analysed. Each analysis cycle lasted for 120 s. Prior to each sample measurement the sensor array was conditioned in honey solution (5 g of honey in 25 mL of distilled water) to obtain stable sensor responses. After every sample measurement a reference sample was analyzed consisting of hydrochloric acid diluted in deionized water (0.01 mol/L) to monitor and correct the drift of sensors in time. The sensors were rinsed with deionized water for 10 s after every analysis cycle. 

Data taken as the average of the last 10 s have been used for further statistical analysis. Moreover, each sample was tested 10 times and the first 6 measurements were discarded, in order to obtain the most stable possible potentiometric signals, according to our previous works [46,47].

### 2.3. Melissopalynological Analysis

The melissopalynological analysis was carried out according to the method UNI 11299:2008, which consists of preparing the microscopic slides with a fixed quantity of honey, followed by the identification and count of the present pollen grains [56]. In particular, 10 g of honey was dissolved into 20 mL of water; the solution was centrifuged at 1000 g for 10 min, and the surnatant was discarded. The residual pellet was suspended in other 20 mL of water, and subjected to a second centrifugation at 1000 g for 5 min; then, the water was decanted. The precipitate remaining at the bottom of the tube was infused with a quantity of glycerin-gelatin, and this material was then transferred onto the glass slide. The slide was covered by a cover slip, for permanent preparation, and heated at 40 °C to allow a homogeneous distribution of the glycerin jelly. The samples were observed under compound microscope with 400x–1000x magnification. Pollen was counted in groups of 100, following parallel equidistant lines uniformly distributed from one edge of the cover slip to the other, until 500 grains had been counted. Therefore, a comparison with the pollen source catalogues of flowers in the study area was performed. For each pollen type the abundance was calculated according to the following equation:

%_p_ = n_p_ × 100/N (1)

where n_p_ is the total number of pollen grains for that particular specie, and N is the total number of all observed pollen grains [57,58,59].

### 2.4. Statistical Analysis

Electronic sensors generate a vast volume of data; therefore, it is necessary to apply methods of data analyses, which allows for data classification [33,34,45,46,47,53,54,55,56,57,58,59,60]. Principal component analysis (PCA) is a dimension reduction technique, which creates a few new variables, called principal components (PCs), from the linear combinations of the original variables, allowing the distribution of samples and variables to be easily plotted and visually analyzed, using the Euclidean distance as a similarity metric [61,62,63]. In order to discriminate between the different honey varieties, a SIMCA (Soft Independent Modeling Class Analogy) method has been developed. This approach is a supervised classification technique that builds a distinct confidence region around each class, after applying a PCA. Then, new measurements are projected into each PCs space that describes a certain class, to evaluate whether they belong to it or not [64]. All statistical analyses were performed using the same native software used for the sensorial analysis (Alpha Soft., Version 12.4., Alpha M.O.S., 2012).

## 3. Results and Discussion

### 3.1. E-Tongue

**Figure 2:**
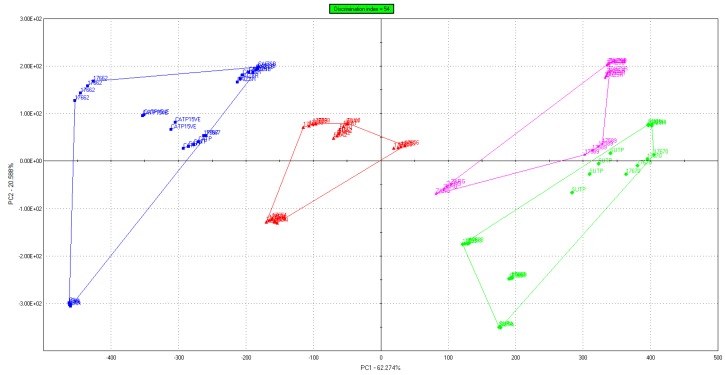
Principal component analysis (PCA) obtained for the different honey varieties. The coloured straight lines indicates the boundaries of each group.

Several authors had already achieved honey classification with different E-tongue technologies [8,12,16,33,43,44,45,46,47,48,49,51,65,66]. Traditional physicochemical parameters used to discriminate between different botanical origins are: (i) electrical conductivity; (ii) mineral composition; and (iii) pH. These properties are related each other [46,67,68,69,70,71]. The ability of the E-tongue to recognize the different honey varieties may arise from the potential measured by the ion-selective electrodes, which is a function of the activity of the ionic species in the honey solution [33,41,46,47,72]. In the present work, the sensor outputs have been used to perform a PCA (Figure 2) with normalized data and 95% of confidence level.

**Figure 3:**
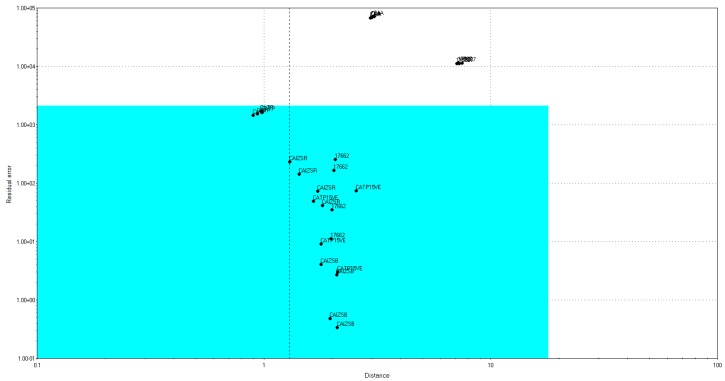
Soft Independent Modeling Class Analogy (SIMCA) model for Chestnut honeys.

The E-tongue proved to be an effective instrument for the discrimination of different honey varieties, with the samples clustered in the bi-dimensional space according to their botanical origins. Later on, the same data matrix has been used to build a SIMCA model with a 90% confidence level, able to recognize the botanical origin of an unknown honey sample. In particular, two samples from each group have been selected as the “training set”, after being authenticated through the melyssopalynological analysis, while the remaining samples have been used as the “testing set”. The four models, one for each honey variety, have been cross-validated, and the corresponding testing set was projected into it. The SIMCA model for the Chestnut honeys revealed that three samples were authentic, while two were multifloral, and positioned outside of the light blue box (Figure 3).

**Figure 4:**
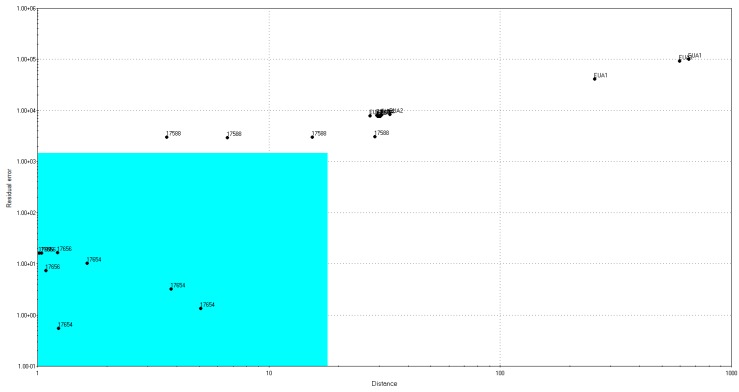
Soft Independent Modeling Class Analogy (SIMCA) model for Eucalyptus honeys.

Regarding the Eucalyptus honeys, all the samples have been classified as multifloral, while none of them was authentic, despite those used as the training set (Figure 4).

**Figure 5:**
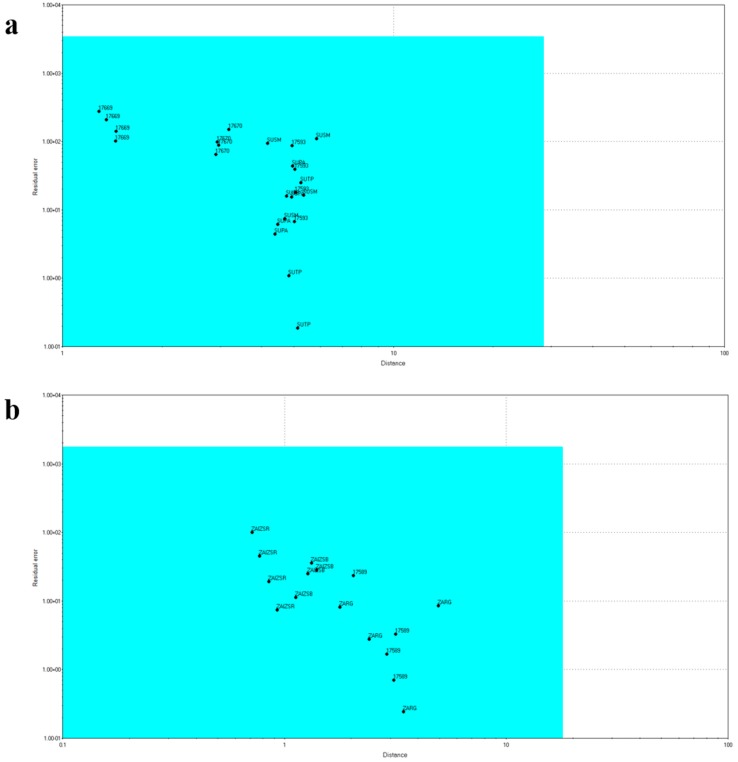
SIMCA model for (**a**) Sulla honeys and (**b**) Citrus honeys.

The opposite result was obtained for Sulla and Citrus honeys, where all the samples have been recognized as authentic (Figure 5a,b).

### 3.2. Melissopalynological Analysis

**Figure 6:**
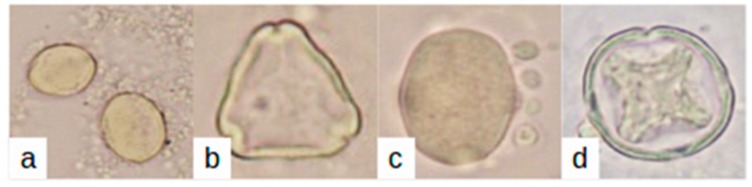
Pollen grains for (**a**) *Castanea*, (**b**) *Eucalyptus*, (**c**) *Hedysarium* and (**d**) *Citrus*.

The photographs of the pollen types, taken under the microscope, are shown in Figure 6.

The results obtained from the melissopalynological analysis for the investigated samples are reported in Table 2.

According to the present results, two samples labeled as “Chestnut honey” were, in reality, multifloral, as the *Castanea* pollen content required to declare a Chestnut honey as unifloral is > 90%. On the other hand, the remaining 5 samples can be considered authentic. The same requirement is stated also for unifloral Eucalyptus honeys, which should contain > 90% of *Eucalyptus* pollen. According to the present results, only two Eucalyptus honey samples can be considered authentic. For Sulla honeys, the percentage of *Hedysarium* pollen required is > 50%; therefore, all the samples are authentic. Regarding the Citrus honey samples, a *Citrus* pollen percentage ≥ 10% is usually required; however, in some circustamces, lower contents are also accepted. In agreement, all the Citrus honey samples were considered authentic.

### 3.3. Validation of E-Tongue Results through Melissopalynological Analysis

A qualitative comparison of the results obtained from E-tongue and melissopalynological analysis is given in Table 3.

As shown, the results are perfectly overlapping. In particular, the melissopalynological analysis revealed that the two Chestnut honey samples, which have been classified as multifloral with the SIMCA model, actually do not meet the requirements in terms of pollen content. Regarding the Eucalyptus honeys, only the two samples used to build the SIMCA model are authentic; therefore the other 4 samples were not recognized as “Eucalyptus” with either of the two techniques. The same results were obtained for Sulla and Citrus honeys, which were all considered authentic by both the analytical methods.

## 4. Conclusions

In this paper, the capability of a potentiometric E-tongue for the classification of Sicilian honeys, according to their botanical origin, was assessed. In addition, a characterization of the honeys’ pollen profiles was carried out through melissopalynological analysis to verify the results achieved by the electronic tongue. The model developed helped to establish limits of acceptability for the membership of a honey to the predetermined category according to the pollen percentage required (Chestnut honey: *Castanea* pollen content > 90%; Eucalyptus honey: *Eucalyptus* pollen > 90%; Sulla honey: *Hedysarium* pollen > 50%; Citrus honey: *Citrus* pollen ≥ 10%). Our work revealed the suitability of the E-tongue in the recognition of honey botanical origins, and its helpful contribution as a rapid and economic support tool for the melissopalynological analysis, which may be used routinely in the future. Specifically, a simple method can be useful for beekeepers to be able to immediately verify the acceptability of a honey and to be able to make quick and useful decisions before labeling.

## Figures and Tables

**Table 1:** Sample set.

Entry	Statistical Analysis label	Declared Botanical Origin	Geographical Origin	Type of Sample
1	CATP15VE	Chestnut	Trapani	Testing
2	17662	Chestnut	Messina	Testing
3	CAA	Chestnut	Catania	Testing
4	CATP	Chestnut	Trapani	Testing
5	CAIZSB	Chestnut	BIPEA Proficiency Testing	Training
6	CAIZSR	Chestnut	BIPEA Proficiency Testing	Training
7	17587	Chestnut	Catania	Testing
8	EUAN	Eucalyptus	Catania	Testing
9	EUA2	Eucalyptus	Catania	Testing
10	EUA1	Eucalyptus	Catania	Testing
11	17656	Eucalyptus	Messina	Training
12	17588	Eucalyptus	Catania	Testing
13	17654	Eucalyptus	Ragusa	Training
14	17669	Sulla	Catania	Testing
15	SUTP	Sulla	Trapani	Training
16	SUSM	Sulla	Agrigento	Training
17	17593	Sulla	Catania	Testing
18	17670	Sulla	Catania	Testing
19	SUPA	Sulla	Palermo	Testing
20	ZAIZSR	Citrus	BIPEA Proficiency Testing	Training
21	ZAIZSB	Citrus	BIPEA Proficiency Testing	Training
22	17589	Citrus	Catania	Testing
23	ZARG	Citrus	Ragusa	Testing

**Table 2:** Pollen analytical data for the investigated samples.

Entry	Predominant Pollen (PP, >45%)	Secondary Pollen (SP, 15–45%)	Important Minor Pollen (IMP, 3–15%)
1	Castanea 93%	Absent	Absent
2	Castanea 92%	<3%	
3	Castanea 72%	Umbelliferae (36%)	Absent
4	Castanea 92%	Absent	Eucalyptus
5	Castanea 93%	Absent	Eucalyptus
6	Castanea >95%	Absent	Absent
7	Castanea 73%	Absent	Hedysarium (14%), Eucalyptus (3.6%)
8	Eucalyptus 69%	Abesent	Hedysarium (11%), Erica (9%), Castanea (3.1%)
9	Eucalyptus 70%	Absent	Hedysarium (13%), Erica (7.5%)
10	Eucalyptus 63%	Hedysarium (16%)	Erica (7.4%)
11	Eucalyptus 92%	Absent	Absent
12	Eucalyptus 79%	Absent	Castanea (8%), Umbelliferae (4.4%)
13	Eucalyptus 95%	Absent	Absent
14	Hedysarium (86%)	Absent	Umbellifearae
15	Hedysarium 89%	Absent	Umbelliferae (3.6%)
16	Hedysarium 91%	Absent	Absent
17	Hedysarium 84%	Absent	Echium (10%)
18	Hedysarium 84%	Absent	Absent
19	Hedysarium (66%)	Absent	Melilotus, Cruciferae
20	Quercus i. (70%)	Citrus (20%)	Oleaceae
21	Citrus 15%	/	
22	Echium (72%)	Absent	Citrus (5.2%), Malus/Pyrus
23	Absent	Absent	Citrus, Hedysarium, Castanea, Echium, Compositae S, Cruciferae, Trifolium

**Table 3:** Comparison of the results from E-tongue and melissopalynological analysis.

Entry	Botanical Origin Confirmed from E-Tongue	Botanical Origin Confirmed from Melissopalynological Analysis	Match between the Two Methods
1	Yes	Yes	Yes
2	Yes	Yes	Yes
3	No	No	Yes
4	Yes	Yes	Yes
5	Yes	Yes	Yes
6	Yes	Yes	Yes
7	No	No	Yes
8	No	No	Yes
9	No	No	Yes
10	No	No	Yes
11	Yes	Yes	Yes
12	No	No	Yes
13	Yes	Yes	Yes
14	Yes	Yes	Yes
15	Yes	Yes	Yes
16	Yes	Yes	Yes
17	Yes	Yes	Yes
18	Yes	Yes	Yes
19	Yes	Yes	Yes
20	Yes	Yes	Yes
21	Yes	Yes	Yes
22	Yes	Yes	Yes
23	Yes	Yes	Yes
